# Reproductive traits and economic aspects on dairy cattle

**DOI:** 10.1590/1984-3143-AR2024-0050

**Published:** 2025-01-31

**Authors:** Jeferson Bello dos Santos, Bruna Waddington de Freitas, Isaac Andres Mora Obando, Natan Dias de Oliveira, Jurandy Mauro Penitente-Filho, Marcus Vinicius Castro Moreira, André Navarro Lobato, José Domingos Guimarães

**Affiliations:** 1 Departamento de Veterinária, Universidade Federal de Viçosa, Viçosa, MG, Brasil; 2 JPF Consultoria, Santa Teresa, ES, Brasil; 3 Programa de Desenvolvimento da Pecuária Leiteira, Universidade Federal de Viçosa, Viçosa, MG, Brasil

**Keywords:** dairy production, economic indicators, reproductive efficiency

## Abstract

This study aimed to characterize the reproductive parameters and economic indicators of dairy farms. Data were from technical assistance of the *Programa de Desenvolvimento em Pecuária Leiteira* (PDPL-UFV) including 26 farms, from September 2022 to August 2023, comprising an entire production cycle. The following economic indicators were selected as dependent variables: Unit Net Margin (UNM), Operating Profitability (OP) and Rate of Return on Capital (RRC). Reproductive parameters were used as explanatory variables in multiple linear regression analysis. A stepwise selection was performed and only variables significant at *p* < 0.10 were kept in the final model. Pregnancy rate of cows, number of inseminations per pregnant heifer, and replacement rate were the reproductive parameters with the greatest effect on the evaluated economic indices. The UNM and OP were positively affected by pregnancy rate of cows and number of inseminations per pregnant heifer, but replacement rate negatively affected both indices. Only the pregnancy rate of cows showed a significant and positive effect on RRC. Results suggest that the economic viability of a dairy farm is not only associated with cutting costs such as reducing the number of inseminations in heifers, or increasing revenue by selling animals, which increases the replacement rate.

## Introduction

Dairy industry plays a fundamental role in the composition of livestock farming in Brazil. According to the 2017 agricultural census, the sector has 1.18 million dairy farms, 216,000 of which are located in the state of Minas Gerais (IBGE, 2019). National milk production reached 23.85 billion liters in 2022, with the state of Minas Gerais being the country's largest producer with 5.86 billion liters (IBGE, 2022).

Reproductive efficiency has the greatest impact on the productivity and profitability of a dairy herd, so that in production systems with inefficient reproductive management there is an increase in involuntary culling, a reduction in the number of animals for replacement and greater expenditure on artificial insemination (Bergamaschi et al., 2010). In addition, there is a reduction in the milk production with economic losses, due to factors such as an increase in the calving interval (Bergamaschi et al., 2010; Marangon-Júnior, 2018), reduction in the proportion of lactating cows per total cows (Lobato, 2009; Marangon-Júnior, 2018), reduction in the conception rate at the first service (Kim and Jeong, 2019), among others.

Due to the close relationship between reproductive traits and economic indicators, both aspects should be considered when evaluating the efficiency of a dairy farm (Bergamaschi et al., 2010; Marangon-Júnior, 2018). It is worth noting, however, that the great socioeconomic and edaphoclimatic diversity that characterizes production systems in Brazil points to the need for regionalized studies (Oliveira et al., 2007).

Therefore, the aim of this study was to characterize the reproductive and economic parameters of dairy farms in the micro-region of Viçosa, state of Minas Gerais. We verified the relationships among reproductive traits and economic indicators such as unit net margin, operating profitability, and the rate of return on capital.

## Methods

### Ethics

The present study was approved by Ethic Committee on Animal Use of the Universidade Federal de Viçosa (CUEA/UFV) under protocol number nº 45/2023.

### Location

Data used in this study are from 26 dairy farms, located in a 60 km radius from the municipality of Viçosa, MG (20.75° S, 42.88° W), including municipalities as Teixeiras, Guaraciaba, Paula Cândido, Coimbra, Guiricema, Visconde do Rio Branco, Cajuri, São Miguel do Anta, Porto Firme and Piranga.

Climate of the region is classified as Cwb by Köppen classification, tropical altitude climate with rainy summers and mild temperatures, with temperatures varying from 13 °C to 30 °C along the year, and monthly rainfall varying from 12 mm to 311 mm (Climatempo, 2024).

### Dataset

We used data generated during technical assistance visits carried out by the *Programa de Desenvolvimento da Pecuária Leiteira* (PDPL-UFV) on 26 farms, from September 2022 to August 2023, comprising an entire production cycle.

The reproductive indicators used were fed with data via Smartmilk (Prodap) zootechnical control software, while the economic indicators were generated using Educampo economic software.

Explanatory variables for analyses are described in Table 1. As dependent variables the following economic indicators were used: Unit Net Margin (UNM; US$/l), Operating Profitability (OP; %), and the Rate of Return on Capital (RRC; %).

**Table 1:** Pre-selected explanatory variables for analyses.

**Explanatory variables**	**Description**
Days to 1^st^ insemination of cows	Time to 1^st^ post-partum insemination; days
Conception in the 1^st^ insemination of cows	Pregnant cows / Inseminated cows in the 1^st^ post-partum insemination; %
Cows with 1^st^ insemination < 90 days	Cows inseminated before 90 days post-partum; %
Cows with 1^st^ insemination > 120 days	Cows inseminated after 120 days post-partum; %
Open days of the cows	Time from parturition to the 1^st^ insemination; days
Interval of days to the cows’ conception	Time from parturition to the 1^st^ fertile insemination; days
Predicted calving interval to the cows	Time from date of the last parturition to predicted date of the next parturition; days
Pregnancy rates of cows	Pregnant cows / Total of cows suitable for insemination; %
Inseminations per pregnant cow	Total of inseminations / Total of pregnant cows; number
Cows with 3 or more inseminations	Cows with 3 or more inseminations / Total of inseminated cows; %
Estrus detection rate of cows	Total of inseminated cows / Total of cows suitable for insemination; %
Age at 1^st^ insemination of heifers	Months
Conception at the 1^st^ inseminations of heifers	Pregnant heifers / Inseminated heifers in the 1^st^ insemination; %
Heifers with 1^st^ insemination < 15 months	Heifers inseminated before 15 months of age / Total of heifers; %
Heifers with 1^st^ insemination > 20 months	Heifers inseminated after 20 months of age / Total of heifers; %
Open months for heifers	Months
Age at 1^st^ calving of heifers	Months
Inseminations per pregnant heifer	Total of inseminations / Total of pregnant heifers; number
Heifers with 3 or more inseminations	Heifers with 3 or more inseminations / Total of inseminated heifers; %
Estrus detection rate of heifers	Total of inseminated heifers / Total of heifers suitable for insemination; %
Replacement rate	%
Primiparous	Total of primiparous / Total of cows; %
Born females	Born females / Total of calves; %
Calving ease	Score 1-4
Dry period	Days
Cows with more than 5 lactations	Cows with more than 5 lactations / Total of cows; %
Heifers older than 12 months	Heifers older than 12 months / Total of heifers; %
Heifers older than 27 months	Heifers older than 27 months / Total of heifers; %

Selection of variables for analyses.

The UNM is the net margin of dairy farming divided by milk production. The UNM was obtained in BRL and converted to USD based on Bid PTAX Dollar in august 31, 2023 [1.0000 USD = 4.9213 BRL; Banco Central do Brasil (2024)].

The OP is the percentage of profit obtained on sales made so it is possible to evaluate the surpluses that the activity allows, providing a parameter for risk analysis.

The RRC indicates the percentage return on the capital invested in the company and, in dairy farming, it is common to use the term rate of return on invested capital, which may or may not include the value of the land (Marangon-Júnior, 2018).In this study, we chose to include the value of the land, which allows us to compare the profitability of dairy companies with those in other regions and/or with other activities.

#### Missing data imputation and evaluation of multicollinearity

Missing data of Conception at the 1^st^ insemination of heifers, Estrus detection rate of heifers, and Calving ease, with one missing value each (3.85%) were imputed by mean imputation.

To evaluate multicollinearity, the explanatory variables were subjected to correlation analysis, so that those with *r* ≥ 0.8 were removed from the analysis. Subsequently, the explanatory variables were assessed for the variance inflation factor (VIF), and those with a VIF value ≥ 10 were removed.

### Statistical analysis

The Statistical Analysis System (SAS OnDemand) was used to analyze the data. After pre-selecting the variables, a multiple linear regression analysis (Reg Procedure) was carried out, using the stepwise selection method, and only variables significant at *p* < 0.10 were kept in the final model. The partial R2 (*partialr2*) was obtained for each explanatory variable through the squared semi-partial correlation coefficient, calculated using the type I sums of squares, using the formula: SS_p_/SS_total_, where SS_p_ is the sum of squares of the parameter, and SS_total_ is the total sum of squares.

## Results

Dataset comprised 26 dairy farms totalizing 2396 cows and 2146 heifers. The description of the variables (economic indicators and reproductive parameters) is shown in Table 2.

**Table 2:** Description of the variables (economic indicators and reproductive parameters) considering the 26 dairy farms used in the study.

**Variables**	**Mean (SD)**	**Median (IR)**
Unit Net Margin (US$/l)	0.10 (0.04)	0.10 (0.07 - 0.14)
Operating Profitability (%)	17.83 (7.79)	18.21 (12.42 - 24.56)
Rate of Return on Capital (%)	8.93 (5.85)	7.92 (5.67 - 14.14)
Days to 1^st^ insemination of cows	72.27 (20.85)	68 (61 - 73)
Conception in the 1^st^ insemination of cows (%)	35.42 (17.14)	32.5 (26 - 40)
Cows with 1^st^ insemination < 90 days (%)	82.15 (14.76)	85.5 (80 - 92)
Cows with 1^st^ insemination > 120 days (%)	9.19 (11.68)	5 (3 - 11)
Open days of the cows	150.04 (25.3)	145 (134 - 167)
Interval of days to the cows’ conception	144.15 (35.14)	145 (127 - 167)
Predicted calving interval to the cows (days)	425.81 (25.2)	422.5 (408 - 444)
Pregnancy rates of cows (%)	13.85 (4.23)	13.5 (12 - 16)
Inseminations per pregnant cow	2.49 (0.77)	2.55 (1.82 - 3.07)
Cows with 3 or more inseminations (%)	39.38 (15.39)	42.5 (35 - 50)
Estrus detection rate of cows (%)	41.27 (11.07)	42 (38 - 48)
Age at 1^st^ insemination of heifers (months)	19.27 (5.06)	17.9 (15.65 - 20.92)
Conception at the 1^st^ insemination of heifers (%)	54.08 (21.19)	50 (40 - 73)
Heifers with 1^st^ insemination < 15 months (%)	25.73 (26.84)	18.5 (0 - 47)
Heifers with 1^st^ insemination > 20 months (%)	34.35 (31.78)	25.5 (10 - 56)
Open months for heifers	22.15 (5.88)	20.39 (18.71 - 24.8)
Age at 1^st^ calving of heifers (months)	28.33 (4.71)	27.4 (24.8 - 30.5)
Inseminations per pregnant heifer	1.92 (0.49)	1.97 (1.55 - 2.31)
Heifers with 3 or more inseminations (%)	22.54 (13.18)	22.5 (14 - 27)
Estrus detection rate of heifers (%)	39.84 (16.49)	40.4 (31 - 51)
Replacement rate (%)	26.00 (10.99)	22.5 (18 - 30)
Primiparous (%)	30.58 (9.42)	29.5 (24 - 35)
Born females (%)	52.36 (12.25)	54.67 (45.16 - 62.07)
Calving ease (1-4)	1.62 (0.73)	1.4 (1 - 1.9)
Dry period (days)	85.04 (19.52)	84 (68 - 94)
Cows with more than 5 lactations (%)	6.57 (6.18)	5.21 (0 - 10.68)
Heifers older than 12 months (%)	59.68 (10.34)	60 (51.52 - 64.86)
Heifers older than 27 months (%)	16.03 (11.74)	13.37 (9.09 - 20.27)

SD, standard deviation; IR, interquartile range. After pre-selecting and evaluating multicollinearity, 16 reproductive traits were used as explanatory variables in the analyses (Table 3).

**Table 3:** Values of VIF for reproductive parameters used as explanatory variables for multiple regression analyses to evaluate the Unit Net Margin, Operating Profitability and Rate of Return on Capital.

**Variable**	**VIF**
Days to 1^st^ insemination of cows	6.33142
Conception in the 1^st^ insemination of cows	3.99674
Open days of the cows	3.38982
Pregnancy rates of cows	3.56266
Estrus detection rate of cows	4.20954
Replacement rate	2.35797
Age at 1^st^ insemination of heifers	4.07293
Conception at the 1^st^ inseminations of heifers	3.46344
Inseminations per pregnant heifer	6.15582
Heifers with 3 or more inseminations	5.19865
Estrus detection rate of heifers	2.42370
Born females	2.75091
Calving ease	1.60625
Dry period	3.85565
Cows with more than 5 lactations	1.46002
Heifers older than 12 months	2.57299

VIF, variance inflation factor.

**Figure 1:**
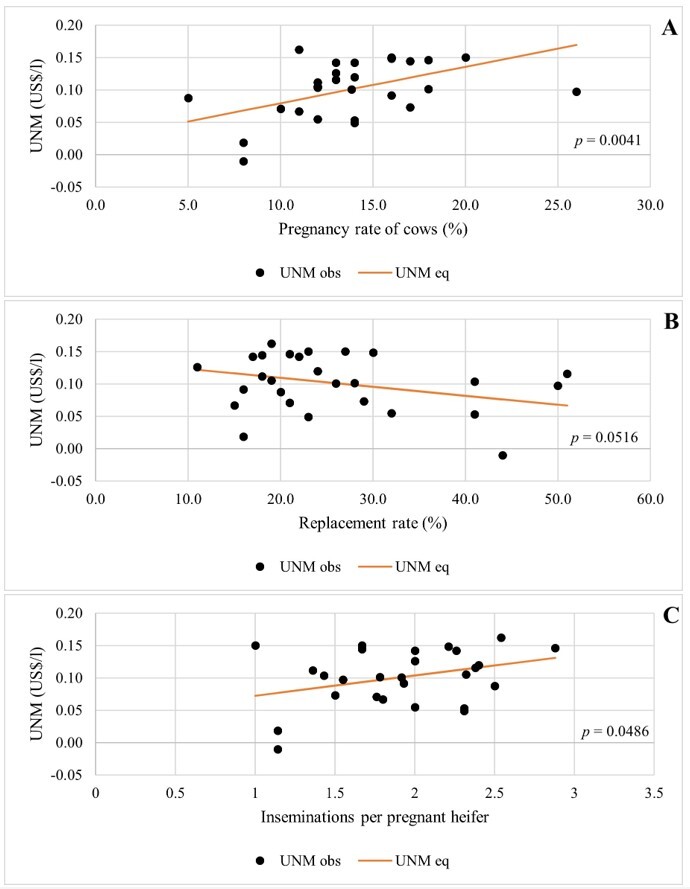
Unit Net Margin (UNM) according to Pregnancy rate of cows (A), Replacement rate (B) e Number of inseminations per pregnant heifer (C). To evaluate one explanatory variable, the others were kept in their average values.

The pregnancy rate of cows presented a positive effect on UNM, so that for every one percentage point increase in the cows' pregnancy rate, UNM increased by an average of 0.006 US$/l (Table 4; Figure 1A). Replacement rate had a negative effect on UNM, and each one percentage point increase represented an average reduction in MLU of 0.001 US$/l (Table 4; Figure 1B). The number of inseminations per pregnant heifer showed a positive effect on UNM, with an average increase of 0.031 US$/l in UNM for each unit increase in the number of inseminations (Table 4; Figure 1C).

**Table 4:** Parameter estimates (±SE) of the multiple linear regression analysis with Unit Net Margin (UNM; US$/l) as dependent variable.

**Parameter**	**Estimate ± SE**	***p*-value**	**Partial R^2^**
Intercept	-0.00044 ± 0.04183	0.9917	-
Pregnancy rates of cows (*x*_1_)	0.00563 ± 0.00175	0.0041	0.17432
Replacement rate (*x*_2_)	-0.00139 ± 0.00068	0.0516	0.13609
Inseminations per pregnant heifer (*x*_3_)	0.03114 ± 0.01491	0.0486	0.11409

SE, standard error; adjusted R^2^ = 0.3460.

Based on the results from Table 4, the equation for UNM is:


$$UNM=-0.00044+0.00563*x_{1}+\left(-0.00139\right)*x_{2}+0.03114*x_{3}$$ (1)

Where $x_{1}$*,*$x_{2}$ and $x_{3}$ are the values of the explanatory variables in Table 4.

**Figure 2:**
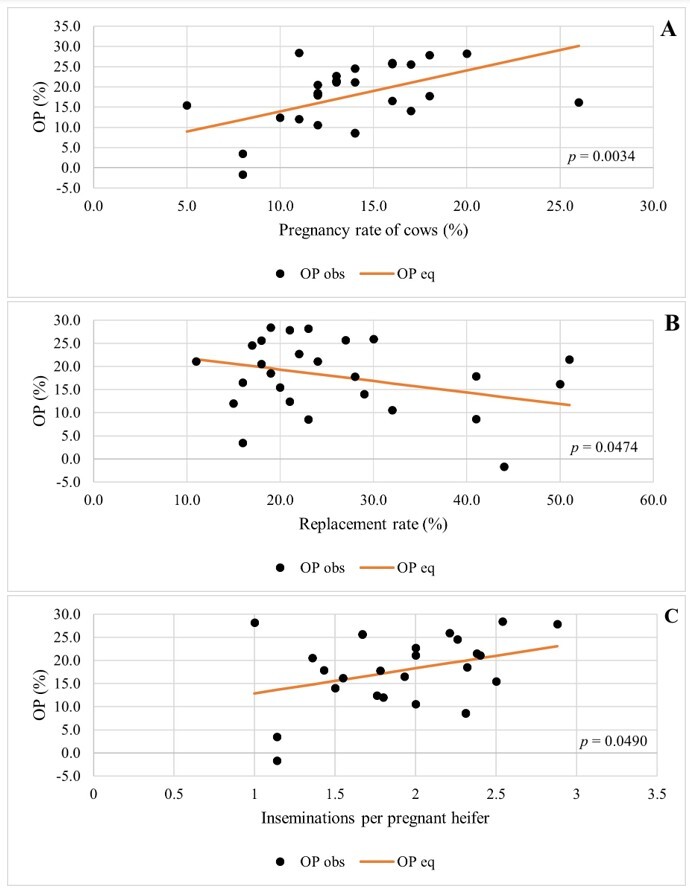
Operating Profitability (OP) according to Pregnancy rate of cows (A), Replacement rate (B) e Number of inseminations per pregnant heifer (C). To evaluate one explanatory variable, the others were kept in their average values.

Results for OP as a dependent variable were similar to those for UNM. The pregnancy rate of cows presented a positive effect on OP, so that for every one percentage point increase in the pregnancy rate, op increased by an average of 1.007 percentage points (Table 5; Figure 2A). The replacement rate had a negative effect on OP, and each one percentage point increase represented a reduction in OP, on average, of 0.249 percentage points (Table 5; Figure 2B). The number of inseminations per pregnant heifer showed a positive effect on OP, with an average increase of 5.429 percentage points in OP for each unit increase in the number of inseminations (Table 5; Figure 2C).

**Table 5:** Parameter estimates (±SE) of the multiple linear regression analysis with Operating Profitability (OP; %) as dependent variable.

**Parameter**	**Estimate ± SE**	***p*-value**	**Partial R^2^**
Intercept	-0.05686 ± 7.30814	0.9939	-
Pregnancy rates of cows (*x*_1_)	1.00740 ± 0.30656	0.0034	0.18157
Replacement rate (*x*_2_)	-0.24867 ± 0.11839	0.0474	0.13916
Inseminations per pregnant heifer (*x*_3_)	5.42939 ± 2.60517	0.0490	0.11200

SE, standard error; adjusted R^2^ = 0.3554.

Based on results from Table 5, the equation for OP is:


$$OP=-0.05686+1.0074*x_{1}+\left(-0.24867\right)*x_{2}+5.42939*x_{3}$$ (2)

Where $x_{1}$*,*$x_{2}$ and $x_{3}$ are the values of the explanatory variables in Table 5.

**Figure 3:**
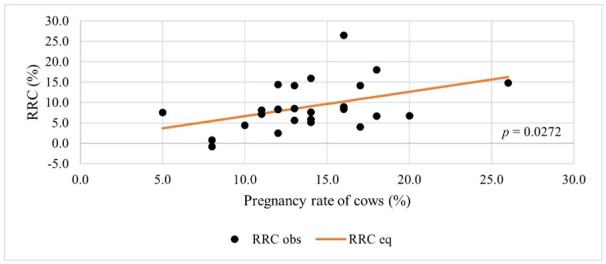
Rate of Return on Capital (RRC) according to pregnancy rate of cows.

Only the pregnancy rate of cows showed significant effect on RRC. For every one percentage point increase in the pregnancy rate of cows, the RRC increased by an average of 0.599 percentage points (Table 6; Figure 3).

**Table 6:** Parameter estimates (±SE) of the multiple linear regression analysis with Rate of Return on Capital (RRC; %) as dependent variable.

**Parameter**	**Estimate ± SE**	***p*-value**	**Partial R^2^**
Intercept	0.63960 ± 3.67948	0.8635	-
Pregnancy rate of cows (*x*_1_)	0.59900 ± 0.25456	0.0272	0.18746

SE, standard error; adjusted R^2^ = 0.1536.

The equation for RRC is:


$$RRC=0.6396+0.599*x_{1}$$ (3)

Where $x_{1}$ is the value of the explanatory variable in Table 6.

## Discussion

The similar results found for UNM and OP are because these variables are related, so that profitability is calculated from the net margin and gross income of the dairy activity. However, only OP is an indicative of risk, because the lower the profitability, the closer the cost is to revenue and the greater the risk of the activity (Marangon-Júnior, 2018).

In the present study, the pregnancy rate of the herds, calculated as the ratio between the number of pregnant cows and the total number of cows suitable for insemination, proved to be the reproductive parameter with the greatest impact on the economic variables, having a significant effect on the three evaluated economic indicators, as well as showing the highest partial R^2^ values. The coefficient of determination (R^2^) shows how much of the total variation is explained by the statistical model, and the partial R^2^ indicates how much each explanatory variable is able to explain of the total variation and is thus a good indication of the importance of each variable on the dependent variable (Marangon-Júnior, 2018).

The pregnancy rate is the result of multiplying the insemination rate by the conception rate (Bergamaschi et al., 2010; Lee, 2011; Pfeifer et al., 2020), which means that it is necessary to increase one of the two factors, or both depending on the farm current situation, in order to obtain an increase in the pregnancy rate. Therefore, it is necessary to inseminate more cows, observing the effectiveness of each insemination. Ideally, the aim is to have over 50% of suitable animals inseminated, with conception above 50%, providing a pregnancy rate above 25%, although higher rates, such as 35%, may be indicated (Bergamaschi et al., 2010).

Insemination and conception rates are dependent on the estrus detection rate, as well as the quality of the semen, the insemination method, and the fertility of the cows (Bergamaschi et al., 2010). Other factors such as body condition score, season, and peri- and postpartum disorders such as dystocia, retained placenta and metritis also influence the pregnancy rate (Kim and Jeong, 2019).

In this study, the factors that compose the pregnancy rate did not show, individually, a significant effect on any of the evaluated economic indicators; however, when they are combined as pregnancy rate, they become the main reproductive variable to be considered, highlighting the multifactorial aspect of fertility in cows.

In the present study, the replacement rate negatively affected UNM and OP. The replacement rate is related to the herd culling rate, which in turn depends on several factors, including diseases in reproductive organs, locomotor and mammary gland problems, advanced age, and marketing (Silva et al., 2008). According to Ribeiro et al. (2003), the rapid replacement of animals in the herd reduces the generation interval and contributes to greater genetic gains. However, milk production depends on the maturity of the cow, and the invested capital needs to be recovered during its productive life. On the other hand, when cows are kept in the herd for too long, milk production and reproduction can be impaired (Hadley et al., 2006).

The culling rate requires a high number of replacement heifers, which increases costs in the rearing phase (Clasen et al., 2024). Besides, reducing the replacement rate can increase the longevity of cows, however, a prerequisite to reduce the replacement rate is a good reproductive performance of the herd and an increase in cow fertility (Clasen et al., 2024).

According to Heikkilä et al. (2008), the optimum point for replacement depends on the cow productive potential. Voluntary replacement is optimal for cows with advanced age and low milk production, and it is more interesting to keep cows with high productive capacity in the herd for as long as possible, up to the tenth lactation. Furthermore, economic cow longevity depends more on cow depreciation than on accelerated genetic improvements in heifers (De Vries, 2017).

In this study, the number of inseminations per pregnant heifer positively influenced UNM and OP. This result shows that, for heifers, it was the insemination rate that brought the economic gain. It is known that increasing the insemination rate the pregnancy rate also increases (Bergamaschi et al., 2010; Lee, 2011; Pfeifer et al., 2020); however, it is common for farmers not to inseminate heifers early, besides it is common to perform only one insemination attempt or use a bull for natural service. This practice reduces the number of inseminations per heifer and may slow the genetic gain, besides increasing the number of male calves born when compared to farms that use sex sorted semen.

Age at first calving is commonly considered as economically optimal when the heifer is 23 to 24 months old at calving (Heinrichs, 1993), and age at first calving before 23 months can be considered advantageous as long as lactation is not compromised (Ettema and Santos, 2004). Nevertheless, efforts to reduce the age at first calving to below 23 months have generally resulted in a drop in milk production in the first lactation (Van Amburgh et al., 1998; Ettema and Santos, 2004), which may be related to the high growth rate during the prepubertal phase that can affect the development of the mammary gland parenchyma (Sejrsen et al., 2000). According to Lin et al. (1988), delaying the first insemination of heifers to reach 26.5 months of age at first calving resulted in increased milk production in the first lactation compared to heifers at 23.3 months of age at first calving, but heifers that calved earlier had higher milk production during the 5-year productive period due to their longer productive life.

It is worth pointing out that the economic viability of the farm is not only associated with cutting costs such as reducing the number of inseminations in heifers, or increasing revenue by selling animals, which increases the replacement rate. Continuous evaluation of the herd reproductive parameters combined with regionalized evaluation of economic indicators can help to find the balance point, maximizing the gains from dairy farming.

## Conclusion

The pregnancy rate of cows, replacement rate, and the number of inseminations per pregnant heifer were the reproductive parameters with the greatest effect on UNM, OP and RRC. Nevertheless, these parameters are dependent on other reproductive parameters, emphasizing the multifactorial aspects of dairy herds fertility.
